# Experiments and Numerical Optimization of Water-Jet Guided Laser Diamond Machining Based on the Improved NSGA-III Algorithm

**DOI:** 10.3390/mi17020206

**Published:** 2026-02-02

**Authors:** Mengjian Wang, Jianwei Wang, Weizhe Wang, Jinhuan Guan, Haoqing Jiang, Hongxing Xu

**Affiliations:** 1 School of Mechanical and Power Engineering, Zhengzhou University, Zhengzhou 450001, China; 2 Institute of Laser Manufacturing, Henan Academy of Sciences, Zhengzhou 450046, Chinahxxu@whu.edu.cn (H.X.)

**Keywords:** water-jet guided laser, diamond, NSGA III algorithm, GPR, uncertainty

## Abstract

This article first investigates the single-factor effects in water-jet guided laser (WJGL) machining of diamond via experiments, analyzing how processing performance responds to laser energy and machining control parameters to define their optimization ranges. Subsequently, an Optimal Latin Hypercube Sampling (OLHD) is adopted to collect experimental data points, enabling exploration of the interaction mechanisms between process parameters and their compatibility with machining performance indicators. A surrogate model based on Gaussian Process Regression (GPR) with combined kernel functions is constructed to capture the complex nonlinear mapping between process parameters and response metrics. To address inherent uncertainties in the optimization model, an improved NSGA-III algorithm integrating the Expected Improvement dominance partition strategy (EIS) is proposed, using Expected Improvement (EI) to determine dominance relationships under WJGL processing uncertainties and derive matched process parameters. Validation via test functions and machining experiments demonstrate that the proposed method outperforms traditional NSGA-III (T-NSGA-III) with significantly lower prediction deviations. The optimized parameters achieved remarkable performance improvements: cutting depth (Nd) increased by 48.21%, kerf width (Kw) reduced by 1.44%, line roughness average (Ra) decreased by 43.09%, and cutting speed (Cs) improved by 78.40%. This research provides a viable process optimization approach for WJGL technology, enabling high-quality, efficient, and robust diamond machining.

## 1. Introduction

Diamond is not only the hardest material in nature, but also possesses ultra-high thermal conductivity, broad-spectrum light transmission, and an extremely low thermal expansion coefficient. These properties make it a core material for high-power laser heat sinks, infrared optical windows, quantum sensor substrates, and ultra-precision cutting tools, supporting the development of strategic fields such as high-end manufacturing and quantum information [1]. However, diamond possesses a three-dimensional covalent bond network composed of high-energy carbon–carbon σ bonds, exhibiting a brittle-ductile transition only at extremely high temperatures—approximately 45% of its melting point [2]. Conventional machining methods such as grinding and polishing [3] rely on microfracture mechanisms for material removal, resulting in surface microcracks, phase transition layers, and edge chipping. These approaches struggle to balance processing efficiency with surface integrity. Various advanced diamond processing techniques have been developed by numerous scholars to overcome the limitations of traditional machining methods, including chemically assisted mechanical machining [4], abrasive water-jet machining [5], focused ion beam [6], electrical discharge machining [7], and laser processing [8]. Among these, laser processing has emerged as a key research focus due to its noncontact nature, flexible machining paths, low equipment costs, and strong process controllability. Material removal in laser processing is achieved by inducing graphitization of diamond materials through high energy density. However, conventional “dry” laser processing generates a larger heat-affected zone [9], recrystallized layers, and thermal damage such as microcracks and debris [10] due to prolonged laser–material interaction time and high energy density. This limits the advancement of laser ultra-precision processing of diamond. To address the above issues, a new WJGL processing technology combining a water jet and a laser was developed by Richerzhagen [11]. This technique utilizes a high-speed water jet to constrain and guide the laser beam, achieving high energy density processing while enabling the water flow to provide efficient real-time cooling and slag flushing of the processing area. Compared to traditional laser processing, it results in a smaller heat-affected zone, cleaner cross-sections, and higher material removal efficiency. Consequently, water-guided laser processing is widely applied to machining hard and brittle materials such as high-hardness alloys [12,13], silicon carbide [14], and cubic boron nitride [15].

Although WJGL exhibits significant advantages in machining hard and brittle materials, its application in single-crystal/polycrystalline diamond processing remains exploratory. Current research primarily focuses on verifying process feasibility [16,17] or conducting single-factor analyses (e.g., the laser power’s effect on quality) [18]. Even promising application studies—such as fabricating complex diamond microchannels for thermal management [17] or achieving high-efficiency micro-tool fabrication [18]—often rely on empirical parameter tuning. However, there is a lack of in-depth research into the quantitative relationships between core process parameters and critical performance metrics, such as kerf width (Kw), notch depth (Nd), and line roughness (Ra).

WJGL processing of diamond involves complex multi-physics coupling. Brecher et al. [19] revealed the dynamic equilibrium relationship between water pressure and laser energy. Spiegel et al. [20] investigated that laser transmission within the water jet exhibits certain attenuation effects. Couty et al. [21] examined the relationship between flow velocity and water-jet stability. Additionally, synergistic optimization of process parameters is essential. Porter et al. [22] studied the influence of varying working distances and feed rates on machining outcomes. Liu [23] investigated how water-jet velocity affects heat-affected zone thickness and material phase transitions on the machined surface. Wang et al. [24] discovered that higher pulse frequencies are not necessarily better. When pulse frequencies are excessively high, molten material and plasma generated are not immediately carried away by the high-velocity water jet. Subsequent pulse energy is absorbed by the plasma, with only a minimal portion acting on the material. This results in low energy utilization per pulse and reduced cutting efficiency.

The aforementioned numerous process parameters collectively form a high dimensional nonlinear response system. These parameter variables include both continuous and discrete types, falling under the category of mixed-variable multi-objective optimization (MVMOO) [25]. Directly conducting process validation would encounter issues such as high costs, high computational complexity, and significant impacts from uncertainties. In engineering practice, experimental design [26] is typically used to collect sample points, followed using surrogate modeling techniques such as polynomial models (PM), Radial Basis Function (RBF), Gaussian Process Regression (GPR), or Support Vector Machine to replace costly physical models. Process parameter optimization is then achieved through multi-objective optimization algorithms. Among these, the GPR model [27] is inherently suitable for small-sample learning and uncertainty quantification. Among multi-objective optimization (MOO) algorithms, Nondominated Sorting Genetic Algorithm III (NSGA-III) [28] maintains the distribution of solutions in high-dimensional objective spaces through its reference point mechanism. Compared with Nondominated Sorting Genetic Algorithm II (NSGA-II) and Multi-objective Evolutionary Algorithm based on Decomposition (MOEA/D), it significantly enhances the ability to approximate the Pareto frontier (PF) [29]. Especially when there are complex trade-off relationships between objectives, NSGA-III can obtain a more accurate Pareto frontier solution (PFS) set through normalized objective space and systematic reference point placement [28]. Therefore, this paper adopts the NSGA-III algorithm for MVMOO of the GPR surrogate model.

However, when optimizing the hybrid variable GPR approximation model using NSGA-III, if the cognitive uncertainty inherent in the surrogate model is not considered [30], the identification of the PFS may be misjudged. Notably, uncertainty is particularly critical in WJGL diamond processing for industrial applications: the process involves complex multi-physics coupling, and subtle variations in key parameters (e.g., ±5% laser power fluctuation, ±0.1 MPa water-jet pressure deviation) can lead to an increase in surface roughness or a decrease in material removal efficiency [31]. Suboptimal solutions within the PFS set may be retained due to coincidentally high evaluation scores, while high-quality solutions may be erroneously eliminated due to noise interference. This ultimately guides the population toward convergence in solution regions with poor actual performance or extreme instability, severely undermining the robustness of the optimization process and the reliability of the results [32]—a consequence that is intolerable in industrial WJGL diamond processing, as it can lead to batch workpiece scrapping and significant economic losses.

Notably, existing uncertainty-aware MOO frameworks exhibit three fundamental limitations that restrict their practical applicability.

(1) They mainly adopt passive noise-resistance strategies, treating uncertainty as post-processing rather than actively coupling uncertainty quantification with the optimization process. (2) They rely on continuous Pareto manifold assumptions, which are incompatible with discrete structural jumps in mixed-variable problems. (3) They fail to balance Pareto frontier improvement and noise suppression under limited samples, often sacrificing solution uniformity or misjudging nondominated relationships.

Representative studies further illustrate these limitations. For instance, Syberfeldt et al. [33] employed dynamic resampling to mitigate noise but provided no mechanism to screen solutions with high improvement potential, thus failing to quantify actual Pareto frontier (PF) advancement. Wang et al. [34] introduced regularity models for noisy optimization, yet their approach relies on the assumption of a continuous Pareto manifold, which is often violated by the structural jumps inherent in mixed-variable problems. Similarly, Rojas-Gonzalez et al. [35] optimized sample allocation via hypervolume divergence but faces challenges with computational intensity, degraded robustness under small samples, and incompatibility with mixed variables.

To overcome these limitations, this study proposes the EIS-NSGA-III (Expected Improvement Partition Screening NSGA-III) optimization framework. Through this EI dominance partitioning and reference-direction-based grouping, the proposed EIS-NSGA-III achieves an effective balance between Pareto frontier improvement, noise suppression, and solution diversity in mixed-variable optimization under limited samples.

In summary, we first conduct single-factor analyses to clarify the influence of key parameters on machining quality and determine the reasonable range of design variables; subsequently, experimental design is employed to acquire sample data correlating process parameters with response indicators, followed by an in-depth analysis of the variable matching relationships between process optimization parameters and between these parameters and machining performance metrics. To address the challenge of obtaining efficient, high-precision process solutions amid high-dimensional nonlinear parameter relationships, a Gaussian Process Regression (GPR) surrogate model is constructed to quantify the intricate correlations between process parameters and machining performance. The EIS-NSGA-III framework is then applied for multi-objective optimization based on the established GPR model. Detailed methods and validation are provided in Section 3. By evaluating candidate individuals based on their uncertainty-aware improvement potential, the framework balances Pareto frontier enhancement, noise suppression, and solution diversity in limited-sample mixed-variable optimization; its accuracy in optimizing WJGL diamond processing parameters and capability to handle uncertainty are further validated, ultimately ensuring the acquisition of process parameters that achieve low damage, deep penetration, high efficiency, and high quality.

## 2. WJGL Processing Experiment

**Figure 1:**
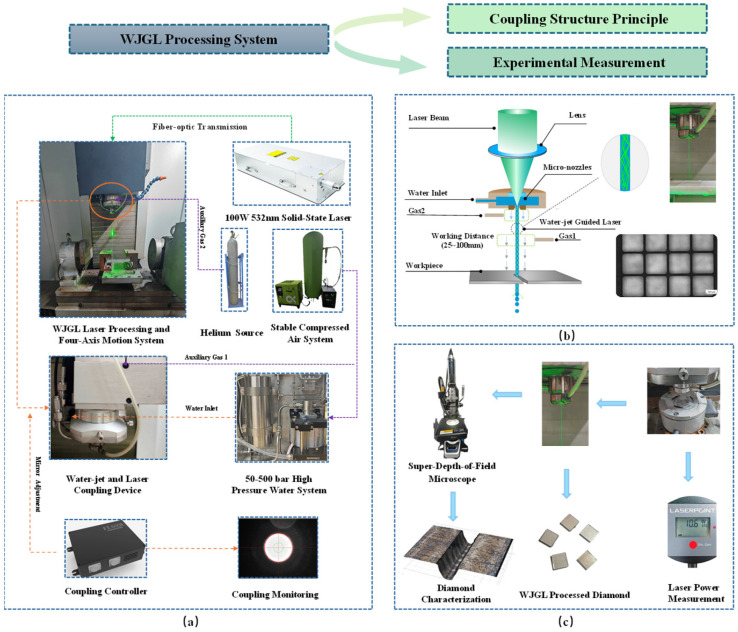
WJGL processed diamond experimental equipment and its principles. (**a**) WJGL processing system. (**b**) WJGL schematic diagram. (**c**) Coupling power and processing measuring device.

This study will conduct single-pass grooving experiments on the surface of diamond pieces to investigate the influence law of water-jet guided laser (WJGL) machining parameters on machining quality. The WJGL diamond processing experimental equipment and its principle used in this paper are shown in Figure 1.

The WJGL equipment (independently developed by our research group) specifically adopts the configuration illustrated in Figure 1a, including a 100 W solid-state laser system operating at a wavelength of 532 nm, a pressurized ultrapure water system with a pressure range of 50–500 bar, a coupling controller, a motion control system with high precision, as well as compressed air and helium gas supply systems. Prior to operation, ultra-pure water achieves stable pressure through an air-driven liquid pressurization system, while helium forms a ring-shaped protective gas via auxiliary gas structure 2 to enhance water-jet stability. The laser is transmitted via optical fiber to the coupling device between the water jet and laser, where the controller aligns the laser spot with the nozzle aperture for precise coupling. During processing, auxiliary gas 1 accelerates to blow away surface water from the workpiece and reduces the impact of water recoil pressure on water-jet stability. The water–light coupling device within the processing equipment and its principle are illustrated in Figure 1b. This configuration consists of a water–laser–auxiliary gas coupling chamber and an 80 μm micro-hole water-jet nozzle. By generating a stable water jet, it facilitates total internal reflection of the laser beam within the jet, thereby realizing precise machining of diamond specimens. Figure 1c depicts the laser power measurement equipment and diamond cutting morphology measurement equipment employed during processing in this experiment.

### 2.1. Single-Factor Analysis of Process Variables

**Figure 2:**
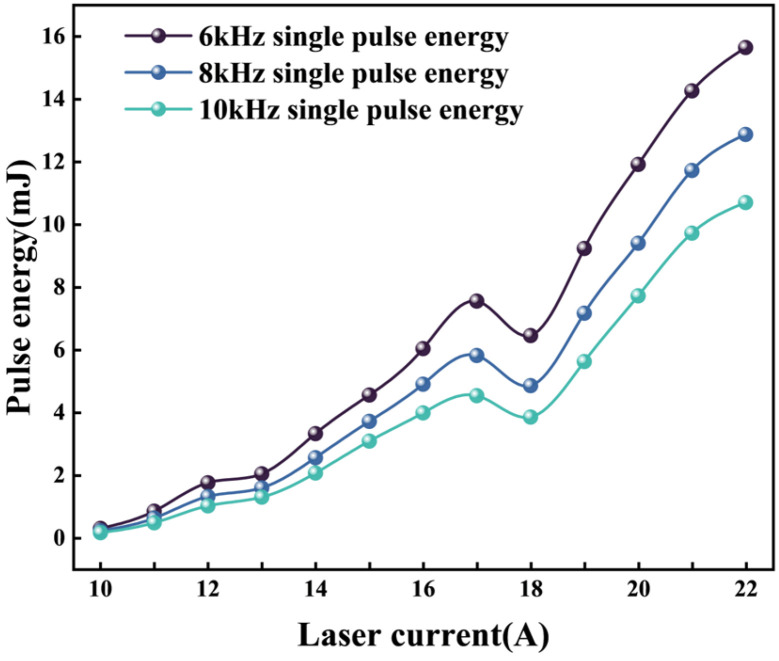
Laser parameter variation and energy relationship curve.

The high-energy YAG laser control system employed in this study regulates power by adjusting the current. The relationship between the laser’s current, repetition rate, and single-pulse energy is illustrated in Figure 2.

Increasing the laser current (Lc) generally shortens the pulse width and boosts the single-pulse energy (observed for 15–17 A in the figure), but a transient deviation occurs at 17–18 A where energy slightly decreases. This nonmonotonic fluctuation stems from the pump threshold effect in the Nd:YAG crystal gain medium: as Lc approaches the stable operating threshold (18 A), pump-induced thermal effects and resonant cavity mode coupling fluctuations weaken population inversion efficiency temporarily. Beyond 18 A, Lc dominant gain overrides these interferences, restoring the linear upward trend of single-pulse energy with Lc. This inflection highlights the nonlinear coupling effects of tuning laser output solely via Lc.

To rigorously isolate the effect of laser energy on processing performance—and to avoid the confounding influences introduced by adjusting either Lc or Lf alone—we employed precise control of the laser’s energy attenuation percentage (Lp). This parameter was regulated via the system’s built-in electro-optical attenuation module, which features a dedicated manufacturer-provided interface allowing direct input of the target attenuation level. The module enables stepless adjustment with an accuracy of ±1%, ensuring that the effective laser energy delivered to the workpiece can be tuned independently while keeping pulse duration, repetition rate, and other temporal characteristics unchanged.

Therefore, although Lc and Lf were selected as primary design variables due to their direct influence on pulse formation, Lp served as a critical secondary control parameter to decouple energy delivery from underlying laser dynamics. This strategy ensures strict adherence to the single-variable principle and provides a more accurate and convincing basis for evaluating the true impact of laser energy on experimental outcomes.

**Figure 3:**
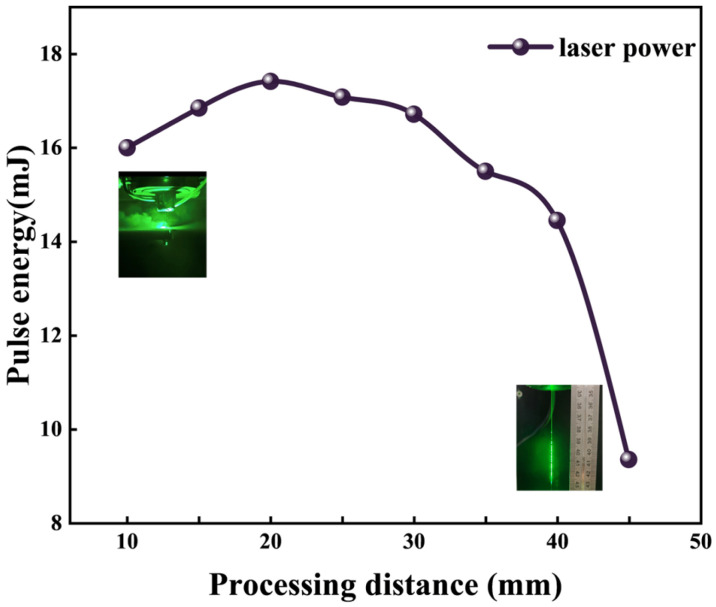
Processing distance and coupling relationship curve.

**Figure 4:**
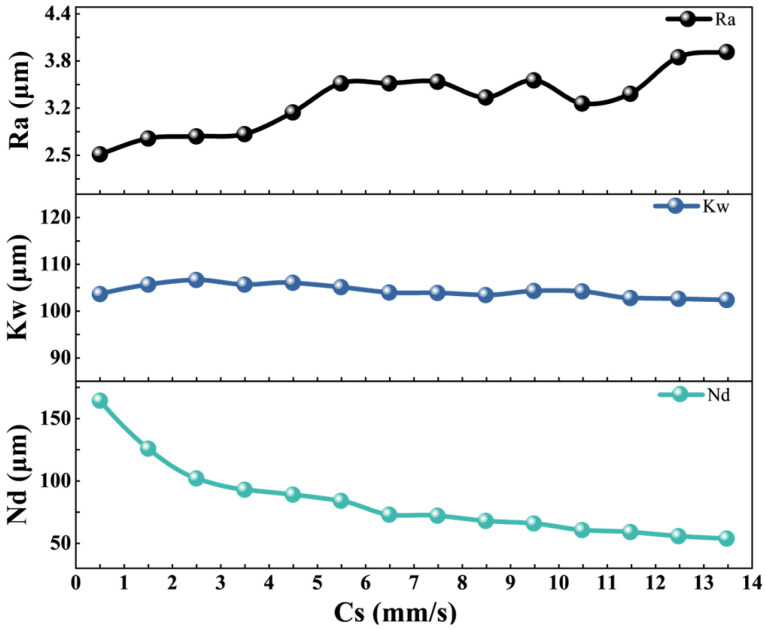
The relationship between cutting speed and cutting quality.

**Figure 5:**
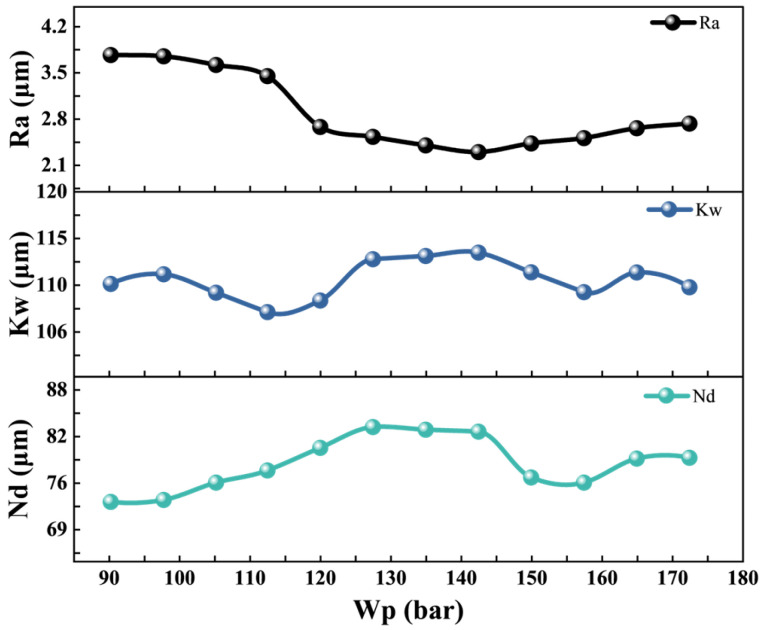
The relationship between water pressure and cutting quality.

To systematically elucidate the influence of key process parameters on the quality of WJGL processing, this study conducted comprehensive experiments investigating the effects of cutting distance (Cd), cutting speed (Cs), and water-jet pressure (Wp). The core findings are summarized in Figure 3, Figure 4 and Figure 5.

First, the effect of the Cd on the effective pulse energy was investigated under constant conditions with a set laser’s energy attenuation percentage of Lp = 80%: Wp = 100 bar, Lc = 18 A, and Lf = 8 kHz.

The nonmonotonic trend of delivered pulse energy with processing distance, as shown in Figure 3, is a direct consequence of two competing physical mechanisms governing the water jet’s stability and its efficacy as a laser transmission medium.

At short processing distances (e.g., 10 mm), the delivered power is sub-optimal not due to inherent laser attenuation in water, but primarily because of hydrodynamic interference. The high-impact interaction between the jet and the workpiece generates intense backspatter and mist. This two-phase flow severely disrupts the laser–water coupling interface at the jet entrance, scattering the incident beam and destabilizing the total internal reflection channel within the jet. Consequently, the coupling efficiency and the effective power transmitted to the workpiece are significantly reduced.

As the distance increases, the spatial separation mitigates this backspatter interference. The water jet develops into a more coherent and stable column, allowing for efficient laser guiding. This explains the observed rise in delivered power with distance in the intermediate range.

However, beyond a critical distance (approximately 45 mm in our setup), the dominant limiting factor shifts from interfacial interference to jet integrity itself. The prolonged free segment of the jet becomes susceptible to breakup. Instability mechanisms—primarily Rayleigh instability (driven by surface tension) and aerodynamic fragmentation (amplified by ambient shear)—overcome the jet’s cohesive forces. The consequent disintegration of the continuous jet into a droplet stream destroys its waveguiding capability. Once the medium becomes discontinuous, the laser beam diverges, leading to the sharp decline in coupling efficiency and delivered power observed at longer distances.

In summary, maintaining the processing distance within an optimal window is critical. This balance minimizes mist interference at short ranges while preventing jet breakup at long ranges, thereby ensuring stable and efficient laser power delivery during machining.

During the cutting process, the water jet cutting speed directly influences the energy interaction time between the jet and the material, as well as the flushing and cooling of the workpiece [24]. In actual manufacturing scenarios, cutting speed directly impacts production efficiency.

Single-pass grooving experiments were conducted on diamond pieces using water-guided laser cutting under a fixed set of parameters: Lc = 19 A, Cd = 20 mm, Wp = 140 bar, Lf = 8 kHz, Lp = 90%. Under these conditions, this study experimentally investigates the effects of cutting speed on Ra, Kw, and Nd, with the results detailed in Figure 4.

All groove quality parameters—line roughness average (Ra), kerf width (Kw), and notch depth (Nd)—were measured using an ultra-depth-of-field microscope. The specific measurement protocols were as follows: Ra was evaluated at the region of maximum line roughness along the groove, with the reported value representing the average of three such measurements at different positions. Kw was determined by averaging width measurements taken at three randomly selected locations. Nd was defined as the maximum cutting depth within the groove, confirmed by three repeated measurements to minimize uncertainty. Throughout this study, except for the final comparative validation where a single representative result is shown for visual clarity, all reported Ra, Kw, and Nd values are averages obtained via this multi-location measurement protocol.

As cutting speed increases, water-jet vibration intensifies and the energy exposure time on diamond material decreases, leading to jet instability and slag residue, which in turn increases Ra. The influence of Cs as a single variable on Kw is negligible. Faster cutting speeds result in shorter laser ablation times, causing Nd to decrease sharply with increasing Cs.

In addition to cutting speed, Wp is another critical parameter that significantly influences processing quality. The effect of Wp on cutting quality was investigated under the following constant conditions: Lc = 19 A, Cd = 20 mm, Cs = 2 mm/s, Lf = 8 kHz, Lp = 90%. Figure 5 illustrates that Wp exerts a significant impact on WJGL processing. When water pressure is moderately increased, the high water-jet pressure enhances the constraint and transmission guidance efficiency of the laser beam while accelerating slag removal. This results in increased Cd and improved surface quality. However, excessively high water-jet pressure violently recoils when the water jet impacts the workpiece surface, generating substantial water mist. This mist scatters laser energy and disrupts stability, degrading laser focusing performance. Consequently, Nd decreases and stability worsens, accompanied by quality issues such as increased roughness and fluctuating Kw.

It should be noted that despite the comprehensiveness of the above single-factor experimental analysis, there are inherent limitations in relying solely on single-factor trends to characterize the actual WJGL processing mechanism. WJGL involves intricate coupled thermo-fluid-optical effects, where multiple process parameters (e.g., laser power, water pressure, cutting distance, and cutting speed) do not act independently but interact dynamically. For instance, the effect of water pressure on laser beam constraint is not fixed; it may be enhanced or weakened under different laser power or processing distance conditions. Single-factor experiments, which fix other parameters while varying only one, can only reveal the individual influence of each parameter but fail to capture such interparameter coupling effects and their synergistic or antagonistic impacts on processing quality (Ra, Kw, Nd). Moreover, single-factor analysis cannot determine the optimal parameter combination that balances multiple conflicting performance metrics (e.g., improving surface quality while ensuring sufficient cutting depth and processing efficiency). These inherent limitations suggest that single-factor results are insufficient to fully guide practical engineering applications. Therefore, it is necessary to conduct surrogate-based multi-objective optimization studies, which can comprehensively consider the coupling relationships among multiple parameters and simultaneously optimize multiple response variables, thereby overcoming the deficiencies of single-factor analysis and achieving efficient and high-quality WJGL processing.

### 2.2. Experimental Design and Parametric Analysis

Based on single-factor analysis, the initial processing parameters for WJGL machining of diamond grooves prior to optimization are shown in Table 1.

**Figure 6:**
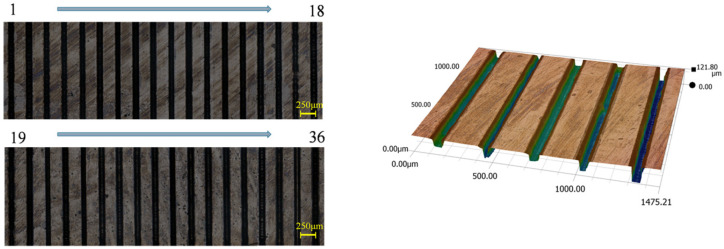
Schematic diagram of slotting experiments on diamond pieces.

To ensure uniformity in the model training samples, the OLHD [36] sampling strategy was employed to sample the design variables. WJGL cutting experiments were conducted, with the experimental results shown in Figure 6.

A deep synthesis of the above results was performed using a super-depth-of-field microscope, and corresponding morphological parameters were measured. This yielded 36 sets of response parameters for Nd, Kw, and Ra, with specific data shown in Table 2.

**Figure 7:**
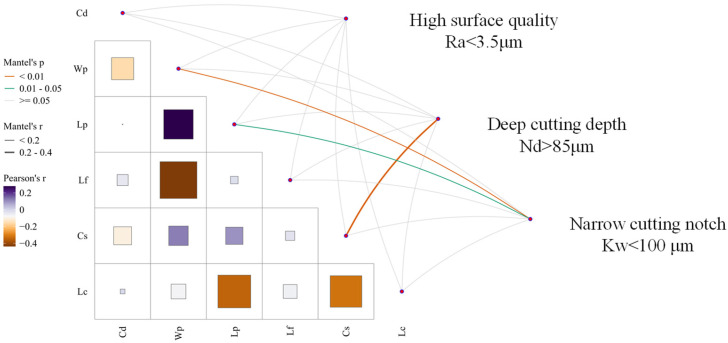
Parameter-associated network diagram.

Based on the 36 sets of complete experimental data in Table 2, this study further employed Pearson linear correlation analysis (Ramsey and Schafer, p. 196) [37] and Mantel multivariate correlation test (Sokal and Rohlf, 2012, p. 852) [38] to construct a correlation network diagram among design variables and a coupling relationship network diagram between design variables and process objectives, as shown in Figure 7.

Figure 7 quantifies the linear correlation strength between design variables using Pearson’s correlation coefficient Pearson’s r. The Mantel test reveals the multivariate coupling patterns between design variables and process objectives (high surface quality, deep cutting depth, narrow cutting notch) through the significance level p and correlation strength Mantel’s r.

Based on the quantitative results in Figure 7, the correlation heatmap reveals complex synergistic and constraining interactions among variables. During WJGL processing of diamond, laser current exhibits a strong negative correlation with Cs, indicating that laser cutting fundamentally involves laser energy acting on the material to achieve ablation removal. The total effective energy absorbed by the material is jointly determined by the energy per unit time and the duration of energy exposure. The strong negative correlation between Wp and Lf reveals that Wp influences the material’s melting state through slag removal and cooling, while Lf controls slag generation rate via energy input frequency. Precise matching between these two parameters is essential for achieving high surface quality machining; otherwise, process failure may occur due to slag accumulation or thermal damage. The process objective–design variable correlation network indicates that some design variables exhibit statistically significant associations with process objectives. For instance, Wp shows a highly significant strong correlation (*p* < 0.01) with deep cutting depth, indicating that high water pressure can rapidly remove cutting slag through strong impact force. This prevents slag accumulation from obstructing laser energy penetration into the material, directly enhancing Nd. Thus, water pressure is the core variable for deep cutting depth. However, some correlations remain insignificant. The *p*-value for high surface quality with other parameters is *p* ≥ 0.05, indicating no statistically significant association. This reflects the “multivariate weak coupling” characteristic of water-guided laser surface quality, where variable interactions are nonlinear, implicit, and mutually masking (individual variables exhibit weak marginal effects, but significant synergistic effects occur in multivariate settings).

Based on the matching relationships and fundamental parameter ranges depicted in the figure, medium-level parameter configurations were selected as the baseline for optimizing the water-guided laser processing of diamond grooving experiments. The initial processing parameters are shown in Table 1.

**Figure 8:**
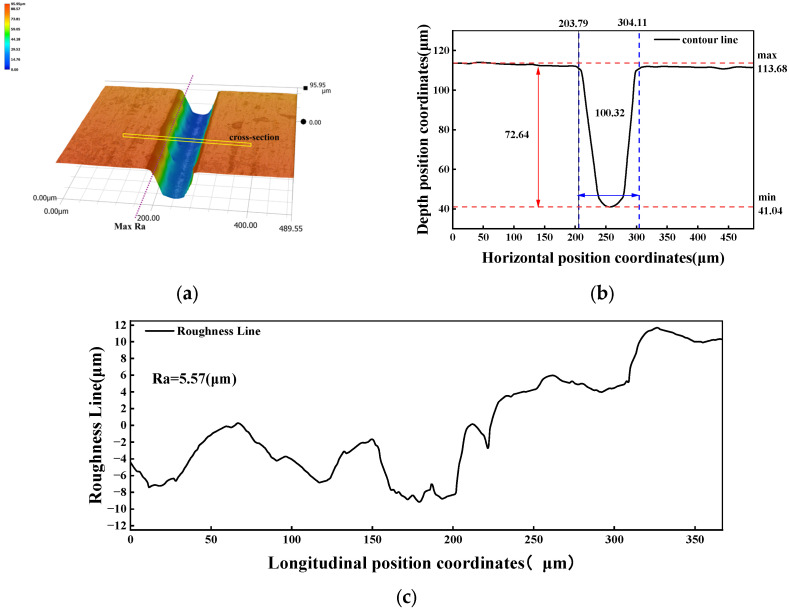
Initial cut quality measurement. (**a**) 3D surface topography of the initial cutting groove. (**b**) Cross-sectional depth profile of the initial cutting groove. (**c**) Roughness profile of the initial cutting surface.

The initial design parameters Kw, Nd, and Ra were measured using the KEYENCE VHX-7000 (KEYENCE Corporation, Osaka, Japan) after machining. The measurement results are shown in Figure 8.

Figure 8a shows the cutting effect after super-depth-of-field depth synthesis. The cross-sectional morphology at the maximum height difference of the cut contour is depicted in Figure 8b, corresponding to a maximum cutting depth of 72.64 μm with a maximum cutting width of 100.32 μm. The maximum Ra after cutting is shown in Figure 8c, with a measured Ra value of 5.57 μm. At a cutting speed of 2.5 mm/s, not only is surface quality suboptimal, but material removal efficiency is also low. Additionally, the cut width exceeds 100 μm, indicating significant room for overall performance improvement. This further demonstrates that while the analysis can qualitatively identify parameter relationships, in the precise optimization of laser cutting processes:

(1) It is challenging to precisely characterize the complex effects of nonlinear interactions among design variables on process objectives, often underestimating the strength of multi-factor coupling.
(2) In small-sample scenarios, the model generalization capability of traditional statistical methods makes it difficult to ensure quality in process optimization. Therefore, to achieve precise prediction, uncertainty quantification, and small-sample robust optimization of laser cutting processes, GPR modeling is required.

## 3. Methods

**Figure 9:**
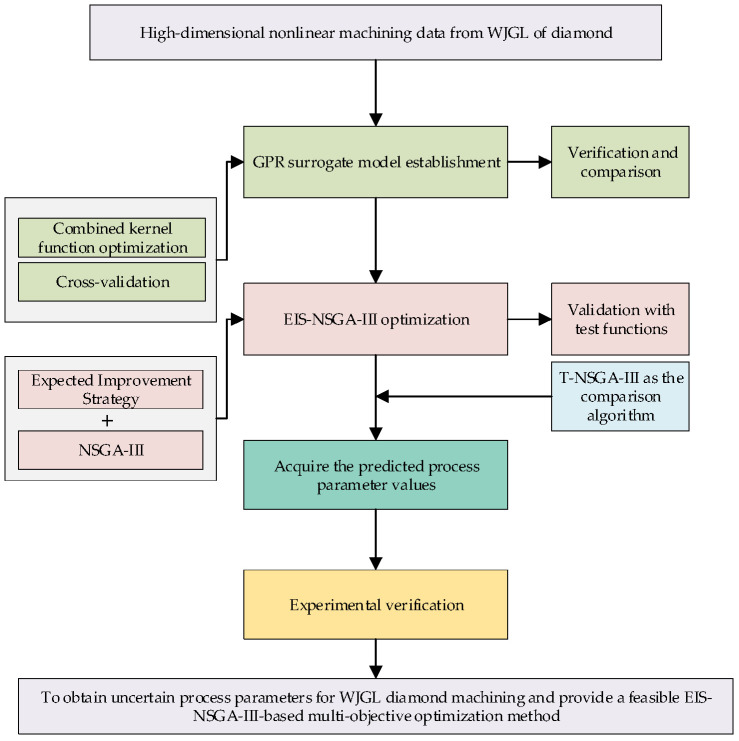
The methodology flowchart.

To address the high-dimensional nonlinear relationships among WJGL machining parameters and the MOO requirement under process uncertainties in Section 2.2, this section proposes a multi-objective optimization method based on Expected Improvement Partition Screening (EIS). The proposed method consists of two core components: surrogate modeling and an improved MOO algorithm. Firstly, the GPR method with composite kernel functions is adopted as the surrogate modeling technique to establish the mapping relationship between process optimization parameters and machining performance response parameters. Secondly, the EIS is introduced to improve the NSGA-III, forming the EIS-NSGA-III algorithm. This improvement aims to overcome the defect of the original NSGA-III in accurately identifying nondominated solutions for uncertain MVMOO models. Specifically, the EI criterion is used to screen nondominated solutions, and reference direction grouping is integrated to ensure the uniformity of dominant solutions, thereby constructing the NSGA-III-based uncertain mixed-variable MOO model with EIS. Prior to its application in WJGL machining optimization, the effectiveness of the EIS-NSGA-III-based model is verified through various MOO test functions. As illustrated in Figure 9 (the methodology flowchart), ultimately forming a complete EIS-NSGA-III optimization model tailored for WJGL diamond machining considering process uncertainties.

### 3.1. Construction of GPR Approximation Models

The GPR surrogate model can quantify uncertainty while outputting predicted values. Its kernel function precisely adapts to nonlinear interactions between variables and enhances model robustness through prior knowledge integration in small sample scenarios, making it a crucial tool for achieving precise optimization of process parameters.

Equation (1) represents the prediction distribution for the *i* sample from the independent GPR model established for the objective function [27].

(1) $$f_{i}(x)\sim GP\left(\mu_{i},\sigma_{i}^{2}\right)$$

In the formula, $\mu_{i}$ represents the GPR predicted mean, while $\sigma_{i}^{2}$ denotes the predicted variance reflecting model uncertainty, with its standard deviation $\sigma_{i}$ defining the individual uncertainty metric.

**Figure 10:**
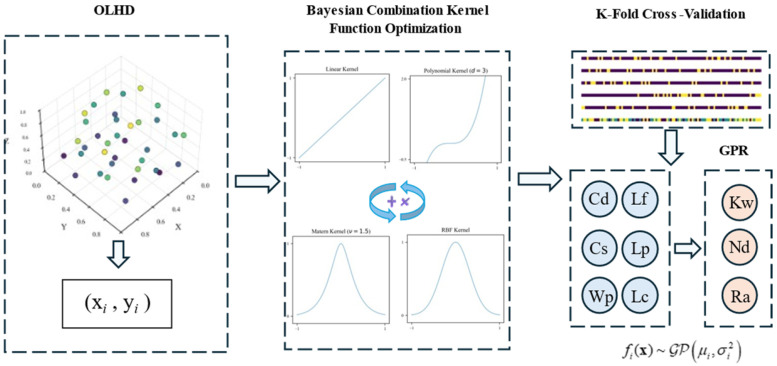
Methodology for Establishing the GPR Model.

As shown in Figure 10, this illustrates the modeling process for a high-precision GPR approximation model using a composite kernel function [39]. For the response indicators and processing parameters obtained from the OLHD sample points, the following key steps are implemented to accurately capture their nonlinear mapping relationships and ensure reproducibility.

A candidate kernel space is constructed, consisting of the Radial Basis Function (RBF) kernel, Matern kernel (ν = 1.5, 2.5), and two types of composite kernels (additive combination of RBF + Matern, multiplicative combination of RBF × Matern). All kernels incorporate a white kernel to quantify measurement noise. Kernel screening adheres to the core criterion of minimizing cross-validation root mean square error (RMSE): RBF is prioritized for smooth response indicators, Matern for nonsmooth responses, and composite kernels for complex nonlinear responses.

Bayesian optimization is employed to optimize kernel hyperparameters (length scale, noise level) and the GPR regularization parameter α (search interval: 1 × 10^−5^ to 1 × 10^−1^, log-uniform distribution). The optimization objective is to minimize the RMSE of five-fold repeated cross-validation (three repetitions), with 25 iterations conducted to balance optimization accuracy and efficiency. Hyperparameter bounds are restricted to physically feasible ranges to avoid meaningless extrapolation.

K-fold repeated cross-validation is used to score and compare different kernel combinations based on RMSE and coefficient of determination (R^2^) [40]. This process identifies the optimal kernel function for each response indicator, enabling the establishment of a high-precision GPR surrogate model with composite kernels.

Additionally, the Gaussian process regression model was compared with the PM response surface model and the RBF model, using the R^2^ as the evaluation criterion. The comparison results are presented in Table 3.

The comparison of R^2^ values among the three models for predicting Kw, Nd, and Ra is shown in Table 3. In Kw prediction, the combined kernel GPR achieved the optimal prediction performance with an R^2^ of 0.977, representing a 4.1% improvement over the second-best RBF model (R^2^ = 0.936). For Nd prediction, GPR achieved an R^2^ of 0.947, slightly lower than the polynomial response surface model’s R^2^ of 0.955 but still demonstrating high accuracy and superior overall performance. Regarding the Ra indicator, both GPR and RBF models achieved an R^2^ of 0.968, showing comparable performance.

**Figure 11:**
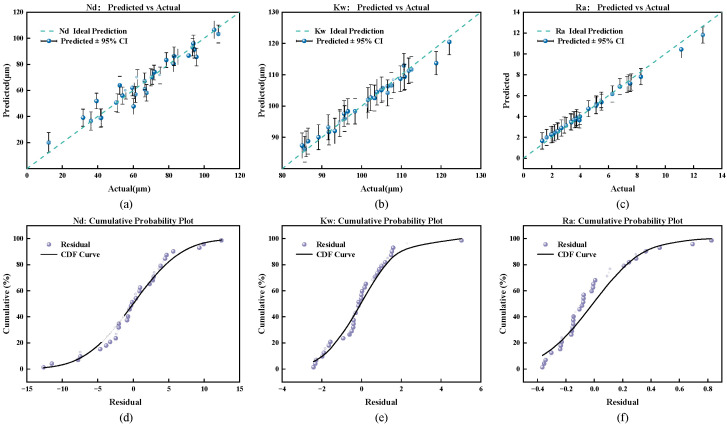
GPR model performance. (**a**–**c**) Predicted vs. actual plots for Nd, Kw, and Ra. (**d**–**f**) Cumulative probability plots of residuals for Nd, Kw, and Ra.

As shown in Figure 11, the fitting performance of the GPR model for water-guided laser processing of diamond is validated from two dimensions: prediction accuracy and residual distribution. Figure 11a–c reveals that the predicted points for Nd, Kw, Ra closely align with the ideal lines. The 95% confidence intervals effectively cover most data points, demonstrating the model’s precise capture of nonlinear relationships and controllable uncertainty. The cumulative distribution function (CDF) plots for the three corresponding parameters in Figure 11d–f show that the residual points for Nd and Kw closely follow the CDF curve with relatively regular distribution. Although Ra’s CDF deviates significantly, indicating slightly higher prediction uncertainty, it still exhibits statistical regularity. In summary, the combined kernel GPR approximation model serves as a reliable surrogate model. However, subsequent optimization should address the uncertainty impact of Ra to ensure the feasibility and stability of process optimization outcomes.

### 3.2. NSGA-III Strategy Based on EIS

A GPR model has been established to map the relationship between process parameters and machining performance in WJGL diamond machining. Although the NSGA-III (as a classic MOO algorithm) is widely adopted for MOO problems, its applicability still needs to be rigorously verified for the WJGL diamond machining scenario, which is subject to significant process uncertainties.

This study uses the Deb–Thiele–Laumanns–Zitzler (DTLZ) test functions [41] to evaluate algorithm performance under uncertainty. However, standard DTLZ functions handle only continuous variables, failing to represent mixed-variable (MV) problems with both continuous and ordered discrete parameters. Therefore, we construct a modified test series, termed MVDTLZ, which incorporates ordered discrete variables while preserving the objective-space characteristics of DTLZ. The variable space is defined as shown in Equation (2):

(2) $$n_{v}=n_{c}+n_{d}$$

where $n_{c}$ is a continuous variable in the range [0, 1], and $n_{d}$ is an ordered discrete variable with values. The objective function calculation remains based on the DTLZ test core formula to simulate the optimization requirements for mixed variables under uncertain conditions.

**Figure 12:**
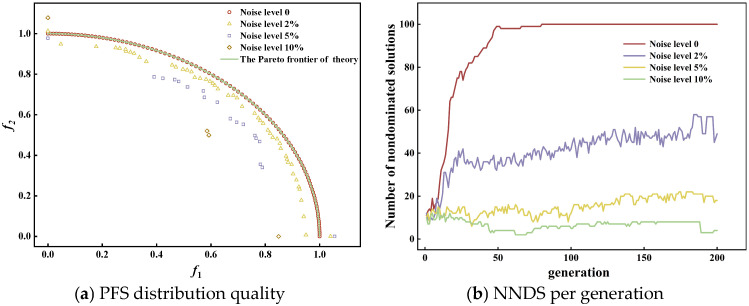
Distribution of PFS under different noise levels.

When optimizing the MVDTLZ2 function without uncertainty, the Pareto frontier (PF) in the objective space exhibits a uniform distribution, as indicated by the ‘○’ in Figure 12a. When uncertainty noise (at 2%, 5%, and 10%) is introduced, fluctuations in the objective function cause the optimized PF to become unevenly distributed. As uncertainty increases, both the distribution quality of the PFS as shown in Figure 12a and the number of nondominated solutions (NNDS) as shown in Figure 12b significantly decrease. Thus, in engineering practice, the NSGA-III algorithm is often constrained by uncertainty, preventing the attainment of highly uniform and accurate PFS.

The reason for the aforementioned defects is that the core advantage of NSGA-III lies in its ability to guide the population to spread over the entire Pareto frontier in the objective space by virtue of pre-set uniformly distributed reference directions, thereby ensuring the uniformity of the obtained solution set. However, process uncertainties inevitably induce deviations between the actual output of the objective function and the predicted values calculated by the algorithm. In addition, the evolutionary operations of NSGA-III (including selection, crossover, and mutation) are all based on the ranking of individual objective function values. Uncertainties can be regarded as adding random noise to the objective function values, making it difficult for the algorithm to distinguish between true performance advantages derived from the intrinsic superiority of individuals and false advantages caused by random noise interference during the selection of elite individuals.

To mitigate the effects of uncertainty, the Expected Improvement (EI) criterion is integrated into NSGA-III. EI, a probability-based sampling function derived from Kriging optimization, balances the exploration of uncertain regions with the exploitation of areas near current optima to guide sampling. The EI calculation [42] for the *i* objective function is shown in Equation (3):

(3) $${\mathrm{EI}}_{i}(x)=\left\{\begin{array}{cc} 0 & \sigma_{i}(x)=0\\ \left(f_{i,min}-\mu_{i}(x))\Phi (z_{i})+\sigma_{i}(x)\phi (z_{i}\right) & \sigma_{i}(x)\neq 0 \end{array}\right.$$

where $\Phi (z_{i})$ and $\phi (z_{i})$ denote the cumulative distribution function and probability density function of the standard normal distribution, respectively, and $z_{i}=(f_{i,min}-\mu_{i}(x))/\sigma_{i}(x)$.

For the multi-objective WJGL processing problem, this paper constructs a weighted EI criterion EI(**x**), as shown in Equation (4):

(4) $$\mathrm{EI}(x)=\sum_{i=1}^{n_{\mathrm{obj}}}w_{i}\cdot {\mathrm{EI}}_{i}(x)$$

where $n_{\mathrm{obj}}$ is the number of objective functions and $w_{i}$ is the weight coefficient.

Based on Equation (4), we propose the Expected Improvement Strategy (EIS). EIS first defines a discrimination criterion for nondominated solutions under uncertainty using the weighted EI (Equation (5)):

(5) $$\mathrm{EI}(a)-\mathrm{EI}(b)>\varepsilon ,\mathrm{Then},(a{≻}_{\mathrm{EI}}b);\mathrm{Otherwise},(b{≻}_{\mathrm{EI}}a)$$

where ε is the improvement potential difference, set to 10^−6^ in this paper. If this condition is not met, the traditional dominance relationship is adopted.

The EIS mechanism primarily addresses model uncertainty. Its core, the weighted EI criterion (Equation (4)), relies on the predicted mean ($\mu_{i}$) and standard deviation ($\sigma_{i}$) from the GPR model, where $\sigma_{i}$ directly quantifies the model’s prediction uncertainty. Furthermore, the discrimination criterion in Equation (5) also operates on this predicted fluctuation range, thereby specifically targeting the reduction of deviations caused by model approximation.

**Figure 13:**
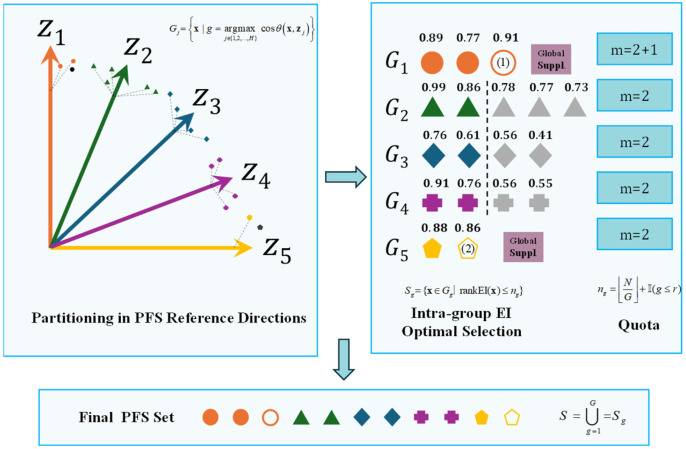
EIS process. Different shapes (circles, triangles, diamonds, etc.) represent solutions from different partition groups (*G*1 to *G*5). Highlighted symbols indicate solutions with high EI rankings, while the markers labeled (1) and (2) denote globally supplementary solutions.

To balance convergence and diversity, an EI screening stratification mechanism was introduced. It ensures that solutions along each reference direction maintain the highest improvement potential, thereby optimizing the overall distribution quality of the Pareto set. This mechanism guarantees that nondominated solutions with uncertainty both approach the theoretical frontier and uniformly cover the entire domain. The process of this mechanism is illustrated in Figure 13.

Firstly, perform reference direction partitioning. Given the target vector $f(x)=\left(f_{1}(x),\ldots ,f_{m}(x)\right)$ of x, and the reference direction $z_{j}$ in the *j* direction, the set *G_g_* is defined as the set of all solutions assigned to the *j* reference direction, as shown in Equation (6):

(6) $$G_{g}=\left\{x\;|\;g=\underset{j\in \{1,2,\ldots ,H\}}{\mathrm{argmax}}\;\cos\theta \left(x,z_{j}\right)\right\}$$

where $cos\theta \left(x,z_{j}\right)$ denotes the cosine similarity for each reference direction, and argmax denotes the maximum cosine similarity value.

Subsequently, quotas are assigned to the partition sets to ensure uniform distribution of PFS subregions. Let *N* denote the total number of selections and *G* denote the number of valid groups. The quota allocation for each partition can be expressed as Equation (7):

(7) $$n_{g}=\left\lfloor N/G\right\rfloor +I(g\leq r)$$

where $\left\lfloor •\right\rfloor$ denotes floor division, r=NmodG represents the number of partitions requiring additional allocations, and I(g≤r) is an indicator function that grants allocations to the first r groups when its condition is true. If some groups fail to meet their quota requirements, the solution with the highest EI value among all unselected solutions is selected to supplement them.

For each partition, the selection process can be expressed as Equation (8).

(8) $$S_{g}=\{x\in G_{g}|\mathrm{rankEI}(x)\leq n_{g}\}$$

For each valid partition $G_{g}$, sort the EI values of all nondominated solutions, denoted as B. The solution set of the final partition is shown in Equation (9).

(9) $$S=\overset{G}{\underset{g=1}{\cup }}=S_{g}$$

### 3.3. Test Function Verification

The proposed method EIS-NSGA-III and T-NSGA-III were compared on the MVDTLZ2 function under 5% uncertainty noise. Algorithm performance was evaluated using a multi-metric system comprising Inverted Generational Distance (IGD) [43], Accuracy of Pareto Set (APS) [35], and Number of Nondominated Solutions (NNDS).

**Figure 14:**
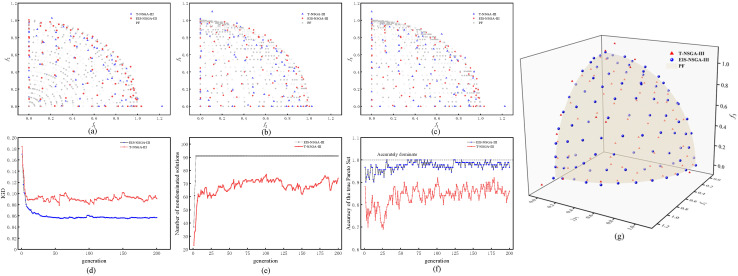
Frontier distribution and metric comparison. (**a**) Projection onto the *f*1-*f*2 plane. (**b**) Projection onto the *f*2-*f*3 plane. (**c**) Projection onto the *f*1-*f*3 plane. (**d**) IGD metric curve. (**e**) Number of nondominated solutions. (**f**) Accuracy of the true Pareto set. (**g**) 3D Pareto front visualization.

IGD measures convergence and diversity via the average distance from the generated set to the true Pareto front. APS quantifies the proportion of true Pareto solutions found. NNDS indicates robustness; a higher count implies better maintenance of solution diversity under uncertainty, offering more options for decision-makers. The PFS distribution and metric comparisons are shown in Figure 14.

By quantifying solution potential and ensuring uniformity via partitioning, this method provides more precise search guidance than traditional NSGA-III. As shown in the two-dimensional projections in Figure 14a–c and the frontier spatial distribution in Figure 14g, this method visually demonstrates that the solutions generated for the MVDTLZ2 problem are closer to the theoretical frontier and more uniformly distributed. Furthermore, the curves of three metrics in Figure 14d–f over generations reveal a decrease in the IGD metric. This indicates that by quantifying improvement potential using the mean and standard deviation of uncertain objectives, the final solution efficiently converges toward the theoretical frontier, resolving the interference issues inherent in traditional methods.

Compared to traditional algorithms, the APS metric exhibits greater stability and approaches 1, while NNDS rapidly stabilizes at 91. This indicates that the EI dominance criterion more accurately distinguishes genuine nondominated solutions from noise-perturbed nondominated solutions. By extending traditional dominance methods to probabilistic improvement-based dominance, it provides more robust solutions for mixed multi-objective optimization under noise, overcoming the shortcomings of conventional approaches that fail to correctly identify dominance relationships in noisy environments. In summary, through its EI-based dominance and partitioning mechanisms, EIS-NSGA-III achieves balanced convergence, accuracy, and distribution uniformity in mixed-variable scenarios, making it suitable for practical engineering problems.

To further validate the accuracy and robustness of the algorithm, the test functions MVDTLZ1-MVDTLZ6 were subjected to 20 independent three-dimensional computations at a 5% uncertainty level, and their mean values were compared. The spacing metric was introduced as a core evaluation indicator. Spacing quantifies the uniformity of solution sets distribution by measuring the standard deviation of distances between solutions and their nearest neighbors. The comparison results are shown in Table 4.

The comparison results demonstrate that the proposed EIS-NSGA-III algorithm exhibits significant advantages across all test functions from MVDTLZ1 to MVDTLZ6.

In terms of comprehensive performance metrics, the IGD was lower than that of the T-NSGA-III algorithm across all problems, with a reduction of 90.3% on MVDTLZ3, further demonstrating its superiority in balancing convergence and diversity. APS improved across the board, particularly in complex problems (e.g., MVDTLZ3) where it rose from 0.629 to 0.994, indicating significantly enhanced accuracy in identifying nondominated solutions. Additionally, the NNDS generated by EIS-NSGA-III consistently reached 91, far exceeding T-NSGA-III ‘s optimal value (81.55), while exhibiting lower overall spacing. This reflects the synergistic enhancement in solution coverage and distribution uniformity. In summary, the proposed algorithm achieves notable improvements in core metrics for multi-objective optimization under uncertainty, demonstrating particularly pronounced advantages in highly complex problems (e.g., MVDTLZ3, MVDTLZ6). This further validates its potential for application in complex engineering scenarios and provides valuable guidance for research on uncertainty-driven process optimization in WJGL diamond processing.

### 3.4. Application to WJGL Diamond Machining Optimization

We apply the EIS-NSGA-III algorithm to solve the multi-objective optimization problem defined in Equation (10):

(10) $$\begin{array}{ll} Decision\;variable & \;x={\left[Cd,\;Cs,\;Wp,\;Lc,\;Lp,\;Lf\right]}^{⊤}\\ \underset{x}{minimize} & \;f(x)={\left[-Kw(x),\;Nd(x),\;Ra(x),\;-Cs\right]}^{⊤}\\ subject\;to & \left\{\begin{array}{l} 0.5\leq Cs\leq 10\\ 90\leq Wp\leq 170\\ 10\leq Cd\leq 35\\ 75\leq Lp\leq 100\\ Lf\in \{6,\;8,\;10\},Lc\in \{\;16,\;17,\;18,19\}\\ Nd\geq 85,Kw\leq 100,Ra\leq 3.5 \end{array}\right. \end{array}$$

The optimization framework imposes constraints on Nd, Kw, and Ra to find the overall best solution. The final Pareto frontier comprises nondominated solutions within the feasible region. To evaluate EIS-NSGA-III on this engineering MVMOO problem under uncertainty, we compare it with T-NSGA-III.

The optimization framework imposes constraints on Nd, Kw, and Ra to find the overall best solution. The final Pareto frontier comprises nondominated solutions within the feasible region. To evaluate EIS-NSGA-III on this engineering MVMOO problem under uncertainty, we compare it with T-NSGA-III. Both algorithms maintain consistent optimization parameters—including population size, crossover and mutation probabilities, and number of iterations—as shown in Table 5.

## 4. Results

**Figure 15:**
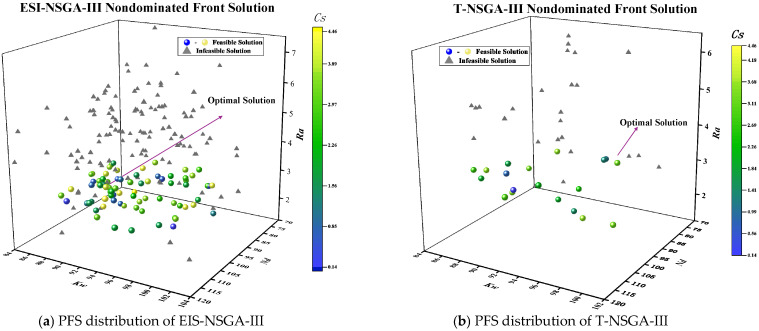
PFS for different methods.

The optimized comparison results are shown in Figure 15.

A comparison of the Pareto frontiers in Figure 15 and the feasible solutions in Table 6 shows that EIS-NSGA-III outperforms T-NSGA-III in solving the uncertain WJGL diamond processing problem. It achieves a superior Pareto set with better convergence and distribution. Specifically, EIS-NSGA-III generates far more Pareto solutions (78 vs. 19), with more uniform and widespread coverage of a superior objective-space region. This demonstrates its enhanced ability to handle constraints and adapt to uncertainty, offering decision-makers higher-quality trade-off solutions.

To balance efficiency and quality, we select the feasible solution with the maximum cutting speed as the optimal process parameter set. Its predicted parameters are listed in Table 6:

The parameter set found by ESI-NSGA-III enables a higher cutting speed (Cs) than that of T-NSGA-III. This advantage stems from better physical matching, as explained below with reference to Table 6 and the relevant figures.

Regarding single-pulse energy (Figure 2), T-NSGA-III uses a 10 kHz pulse frequency (Lf), which yields lower energy per pulse than the 8 kHz used by ESI-NSGA-III at comparable currents. This forces T-NSGA-III to adopt a lower cutting speed (3.40 mm/s) to avoid incomplete ablation [33]. In contrast, the 8 kHz Lf of ESI-NSGA-III, paired with a 19 A laser current (Lc), provides sufficient pulse energy to support a higher speed (4.46 mm/s).

**Figure 16:**
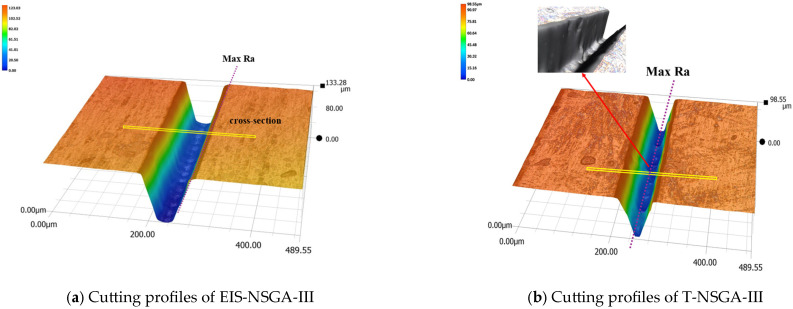
Comparison of cutting profiles between two algorithms.

The process parameters from Table 6 were experimentally validated via actual WJGL diamond cutting. The resulting cutting profiles are compared in Figure 16.

Figure 16a shows diamond cutting using process parameters optimized by the EIS-NSGA-III algorithm. The diamond groove exhibits smooth walls with uniform roughness distribution in the Max Ra region. In contrast, Figure 16b displays groove walls processed with parameters optimized by T-NSGA-III, revealing defects such as pronounced recast layers and poor surface integrity.

**Figure 17:**
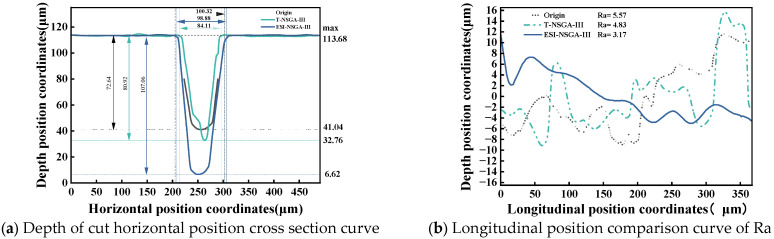
Schematic diagram of cut cross section and contour line at Max Ra.

To further analyze the accuracy of both algorithms and the machining quality, cross-section and Max Ra region contour plots were constructed for the two algorithms, as shown in Figure 17:

Figure 17 shows that all result indicators of both EIS-NSGA-III and T-NSGA-III are superior to the initial ones. Although the Kw value of T-NSGA-III exhibits the best performance among the three, being only 84.11 μm, the predicted results of this algorithm have deviated due to the accumulation of uncertainty deviations. The obtained solutions, as shown in Table 7, exhibit large predictive deviations, with all response parameters exceeding 14.34% and failing to meet the constraints of feasible solutions. In contrast, the deviations of EIS-NSGA-III are all below 9.46% and all satisfy the constraints of feasible solutions.

Further analysis combining the contour lines with the T-NSGA-III design variable parameters in Table 6 reveals that optimization based on this optimal solution suffers from issues such as low laser beam energy and extended processing distance. This results in the formation of a remelted layer on the cut seam contour, exhibiting significant nonuniformity, as shown in Figure 17a,b. The groove sidewalls and bottom exhibit poor morphological consistency, failing to meet the constraints for maximum cutting depth Nd and Ra. Consequently, the processing quality falls short of the EIS-NSGA-III optimized results, which achieved a maximum depth of 107.06 μm and a minimum Ra of 3.17 μm.

In summary, this paper employs the ESI-NSGA-III algorithm to obtain the final optimized process parameters for a single diamond grooving experiment. Compared to the T-NSGA-III algorithm, which exhibited constraint violations and prediction errors exceeding 14.34%, the ESI-NSGA-III algorithm achieved prediction errors below 9.46% in its final optimization. The maximum cutting depth increased by 48.21% compared to the initial results, while the cutting width decreased by 1.44%, roughness reduced by 43.09%, and cutting speed improved by 78.40%. This high efficiency directly translates into reduced consumption of costly auxiliary gases like helium, lowering operating expenses.

This demonstrates that the ESI-NSGA-III algorithm effectively reduces the sensitivity of optimization results to uncertainty, ensuring that the optimized parameters retain a certain degree of accuracy when applied to engineering practice, ultimately yielding a high-precision optimal solution. The EIS strategy core to this algorithm framework is generalizable, indicating its strong potential for extension to the optimization of laser processing for other hard and brittle materials (e.g., silicon carbide), thereby enhancing the practical relevance and scope of this methodology.

## 5. Conclusions

### 5.1. Summary

In this work, a multi-parameter OLHD was adopted to construct a composite kernel function GPR surrogate model, which effectively quantifies the nonlinear interactions between design variables and process parameters in WJGL diamond machining. To address the practical challenge of parameter optimization under machining uncertainties, the EIS-NSGA-III algorithm was further employed. This integrated approach ultimately yields high-quality, high-efficiency, and robust WJGL processing parameters, providing a reliable technical reference for industrial diamond machining.

(1) Single-factor experiments revealed fundamental trade-offs: laser power primarily drives Nd but compromises Ra and Kw; Wp optimizes cooling and guidance within a critical range; and cutting speed directly trades off Nd against Ra. The subsequent multi-objective optimization, guided by the criterion of maximizing Cs while satisfying thresholds for Kw and Ra, successfully identified a Pareto-optimal set of parameters, providing a principled solution space for balancing competing objectives in industrial applications.
(2) Experimental design was employed to obtain sample points and analyze the nonlinear interactions between process parameters. Consequently, a GPR surrogate model with a combined kernel function was established for each response parameter. The impact of model uncertainty on optimization results was analyzed, revealing that nondominated relationships became difficult to capture when uncertainty reached 10%. To address this, an EIS model was established to identify nondomination relationships among uncertain manufacturing process parameters during multi-objective optimization.
(3) Post-optimization experimental validation demonstrates that the EIS-NSGA-III algorithm better captures the nonlinear relationships among variables, achieving higher optimization accuracy than the T-NSGA-III algorithm for process parameters: its prediction deviations for all metrics were below 9.46%, significantly outperforming the T-NSGA-III algorithm with deviations exceeding 14.34%, which demonstrates that the EIS algorithm effectively reduces the interference of uncertainty in the optimization process compared to T-NSGA-III.
(4) The optimized WJGL diamond pieces processing parameters were obtained as follows: Nd increased by 48.21%, Kw decreased by 1.44%, Ra reduced by 43.09%, and Cs improved by 78.40%. This research provides a feasible, high-precision, high-quality, and high-efficiency optimized process parameter set and optimization method for WJGL diamond processing.
(5) Implications for practical applications: the primary practical value of this work lies in providing a systematic, data-driven methodology to replace costly and time-consuming trial-and-error in industrial process development. The obtained Pareto-optimal solutions cater directly to diverse manufacturing scenarios.

### 5.2. Limitations and Future Work

While the EIS-NSGA-III algorithm demonstrates superior performance and robustness in optimizing the WJGL process for diamond grooving, this study has several limitations that should be acknowledged.

(1) The experimental validation was confined to a specific type of single-crystal diamond and a fixed groove geometry, thus limiting the generalizability to other diamond grades or complex workpiece geometries.
(2) The incorporation of physical constraints and uncertainty quantification, though critical for robustness, results in increased computational overhead.
(3) Direct application of the framework to other materials (e.g., silicon carbide) or different WJGL configurations would require the recalibration of material-specific physical models.

To address these limitations and further enhance the framework’s practicality, future research will be directed along three key pathways: (i) extending the methodology to multi-pass and 3D machining scenarios for fabricating complex geometries; (ii) exploring strategies for real-time adaptive optimization to dynamically compensate for process variations; and (iii) developing a modular and material-agnostic framework to facilitate efficient adaptation to a wider range of materials and machine configurations, thereby accelerating broader industrial adoption.

## Figures and Tables

**Table 1:** Design variable initialization and range.

Design Variable	Initial Value	Range
Cd (mm)	25.00	10–35
Wp (bar)	110.00	90–170
Lp (%)	80	75–100
Lf (kHz)	8	6, 8, 10
Cs (mm/s)	2.50	0.5–10
Lc (A)	18	16, 17, 18, 19

**Table 2:** Design variables and response parameters.

	Cd (mm)	Wp (bar)	Lp (%)	Lf (kHz)	Cs (mm/s)	Lc (A)	Nd (μm)	Kw (μm)	Ra (μm)
1	23.39	90.2	75.79	10	4.11	18	91.26	91.76	3.75
2	27.13	93.07	87.14	10	1.02	18	105.64	106.55	6.27
3	24.76	96.03	89.99	10	8.64	16	55.86	89.14	4.59
4	30.89	96.99	79.93	8	6.78	18	39.3	94.51	5.49
5	21.66	100.6	79.37	10	8.9	16	50.38	86.04	8.28
6	32.58	104.87	82.25	8	5.12	17	66.57	93.26	3.75
7	29.71	108.16	87.66	10	2.88	17	85.21	85	1.31
8	20.96	111.16	95.14	6	8	19	53.98	109.76	2.06
9	22.84	114.93	99.74	10	6.02	16	71.95	105.05	5.12
10	18.68	118.06	98.95	6	1.63	16	108	94.5	2.2
11	28.89	119.42	91.09	8	8.15	18	60.22	101.41	7.53
12	34.04	121.34	82.68	8	1.53	19	95.64	95.72	5.17
13	33.23	123.57	77.24	6	7.64	16	36.14	95.3	3.98
14	26.19	126.11	83.84	8	4.53	19	71.72	91.6	7.43
15	19.82	133.28	93.2	6	3.7	17	78.66	101.58	3.7
16	28.35	140.8	75.62	6	1.89	17	83	98.36	6.8
17	34.88	143.48	98.57	6	7.35	17	12.33	86.51	13.61
18	24.14	147.32	95.58	6	0.77	17	93.5	107.49	9.65
19	31.6	149.21	96.15	10	2.27	19	52.43	103.25	7.61
20	19.46	150.61	90.93	10	8.97	16	61.26	111.87	3.34
21	27.79	157.48	96.73	10	6.04	16	67.52	112.52	2.44
22	25.8	164.49	84.05	6	9.27	19	41.62	102.3	2.68
23	22.03	167.8	94.27	8	7.04	16	60.55	122.09	1.33
24	30.47	167.83	85.46	6	2.45	19	69.93	106.57	3.54
25	15.6	101.02	81.25	8	0.63	19	121.67	85.36	3.01
26	13.44	107.72	88.56	10	6.38	18	75.16	103.86	2.88
27	15.47	113.32	77.07	10	2.72	18	93.93	85.72	5.5
28	17.44	131.39	84.77	8	5.38	18	70.69	118.77	3.95
29	14.17	135.43	97.58	8	9.77	17	51.23	110.7	3.38
30	17.86	137.41	77.81	8	5.55	19	59.51	110.64	1.64
31	12.62	143.28	91.69	10	3.34	18	94.54	96.53	7.31
32	11.31	154.42	78.76	6	7.5	18	41.91	105.34	5.13
33	16.73	156.17	92.67	6	3.57	17	82.76	108.09	2.39
34	11.36	159.68	86.78	8	4.26	17	62.33	110.85	11.13
35	13.7	161.79	89.44	6	9.73	16	31.74	95.61	3.34
36	10.63	128.83	81.45	8	4.77	19	66.42	104.94	1.96

**Table 3:** Comparison of R^2^ values among three approximation models.

Approximate Modeling Method	Kw R^2^	Nd R^2^	Ra R^2^
PM response surface	0.821	0.955	0.886
RBF	0.936	0.903	0.968
GPR	0.977	0.947	0.968

**Table 4:** Comparison of metrics for different test functions.

Test Function	Algorithm	IGD	APS	NNDS	Spacing
MVDTLZ1	T-NSGA-III	0.048	0.980	81.550	0.074
	ESI- NSGA-III	0.021	0.997	91.000	0.056
MVDTLZ2	T-NSGA-III	0.090	0.876	68.791	0.032
	ESI- NSGA-III	0.056	0.985	91.000	0.030
MVDTLZ3	T-NSGA-III	0.580	0.629	37.560	0.924
	ESI- NSGA-III	0.056	0.994	91.000	0.144
MVDTLZ4	T-NSGA-III	0.282	0.797	68.513	0.044
	ESI- NSGA-III	0.273	0.981	91.000	0.033
MVDTLZ5	T-NSGA-III	0.083	0.845	71.873	0.036
	ESI- NSGA-III	0.057	0.992	91.000	0.035
MVDTLZ6	T-NSGA-III	0.396	0.851	70.716	0.354
	ESI- NSGA-III	0.079	0.998	91.000	0.099

**Table 5:** Optimized parameter settings.

Population Size	Iteration Count	Crossover Probability	Mutation Probability	Discrete Variable Mutation	Continuous Variable Mutation	Reference Direction
200	400	0.9	0.3	Neighborhood	Polynomial	200

**Table 6:** Parameters corresponding to optimal speed for two algorithms.

Algorithm	Cd	Wp	Lp	Lf	Cs	Lc	Nd	Kw	Ra	NNDS	Feasible Solution
T-NSGA-III	28.23	112.29	82.33	10	3.40	17	91.81	97.79	3.41	44	19
ESI-NSGA-III	16.34	104.43	98.25	8	4.46	19	117.67	94.50	3.47	200	78

**Table 7:** Predictive experimental verification.

Comparison Items	Nd	Prediction Deviation	Kw	Prediction Deviation	Ra	Prediction Deviation	Feasible Solutions
Initial Results	72.64	-	100.32	-	5.57	-	-
T-NSGA-III	80.29	14.34%	84.11	16.26%	4.83	29.40%	NO
ESI-NSGA-III	107.66	9.29%	98.88	4.43%	3.17	9.46%	YES

## Data Availability

The original contributions presented in this study are included in the article. Further inquiries can be directed to the corresponding authors.

## Abbreviations

The following abbreviations are used in this manuscript:

WJGL	water-jet guided laser
EI	expected improvement
EIS	expected improvement partition screening
NSGA-III	nondominated sorting genetic algorithm III
MVMOO	mixed-variable multi-objective optimization
MOO	multi-objective optimization
RBF	radial basis function
PM	polynomial models
GPR	gaussian process regression
T-NSGA-III	traditional NSGA-III
EIS-NSGA-III	expected improvement partition screening NSGA-III
NNDS	number of nondominated solutions
PF	pareto frontier
PFS	pareto frontier solution
IGD	inverted generational distance
APS	accuracy of pareto set
Cs	cutting speed
Ra	line roughness average
Kw	kerf width
Nd	notch depth
Lp	laser’s energy attenuation percentage
Lf	laser repetition rate
Wp	water-jet pressure
Cd	laser cutting distance
DTLZ	Deb–Thiele–Laumanns–Zitzler
R^2^	coefficient of determination
OLHD	optimal latin hypercube experimental design
